# Euglycemic Diabetic Ketoacidosis Triggered by Sepsis in a Patient on Sodium-Glucose Co-transporter 2 (SGLT2) Inhibitor Therapy: A Case of Diagnostic and Therapeutic Challenges

**DOI:** 10.7759/cureus.87029

**Published:** 2025-06-30

**Authors:** Archana Ramalingam, Ayele Tewadros, Bruno De Souza Goncalves, Anitha Yelangi, Ashraf Elghul

**Affiliations:** 1 Internal Medicine, New York Medical College/Saint Clare's Health, Valhalla, USA; 2 Department of Cardiology, St. Mary’s Medical Center, Huntington, USA; 3 Cardiology, University of Iowa Hospitals and Clinics, Iowa City, USA; 4 Biomedical Sciences, Marshall University Joan C. Edwards School of Medicine, Huntington, USA; 5 Department of Endocrinology, Hershel Woody Williams VA Medical Center, Huntington, USA; 6 Internal Medicine, Marshall University Joan C. Edwards School of Medicine, Huntington, USA

**Keywords:** euglycemic diabetic ketoacidosis, ketosis, sepsis, sodium-glucose co-transporter 2 inhibitor, type 2 diabetes mellitus

## Abstract

Euglycemic diabetic ketoacidosis (EDKA) is an uncommon life-threatening condition increasingly recognized in patients treated with sodium-glucose co-transporter 2 (SGLT2) inhibitors. Its diagnosis is often delayed due to the absence of significant hyperglycemia. Sepsis can further complicate the clinical scenario, enhancing insulin resistance and promoting ketoacidosis. We report the case of a 69-year-old male with a history of diabetes mellitus managed with insulin, semaglutide, and empagliflozin, who presented with syncope, bradycardia, and gastrointestinal symptoms following antibiotic treatment for a lower extremity infection. Laboratory findings revealed high anion gap metabolic acidosis, lactic acidosis, mild hyperglycemia, and methicillin-resistant Staphylococcus aureus (MRSA) bacteremia. Despite unremarkable imaging, suspicion for EDKA was heightened due to severe metabolic derangements and the patient’s SGLT2 inhibitor use. Multidisciplinary management included aggressive fluid resuscitation, insulin therapy, empirical antibiotic coverage with vancomycin, and surgical debridement of a subsequently identified foot abscess. Clinical improvement paralleled the resolution of metabolic acidosis and bacteremia. This case highlights the critical need for early recognition of EDKA in patients on SGLT2 inhibitors, particularly in sepsis. Timely diagnosis, prompt management of underlying infections, and a multidisciplinary approach are essential for favorable outcomes.

## Introduction

Euglycemic diabetic ketoacidosis (EDKA) is a serious metabolic complication characterized by metabolic acidosis with an excess production of ketone bodies, without marked hyperglycemia [1]. Although the incidence of diabetic ketoacidosis (DKA) is well-established, with approximately 4.6-8 episodes per 1,000 patients with diabetes annually, the prevalence of EDKA remains underestimated due to diagnostic challenges [1,2]. Recent studies suggest that around 2.6% to 3.2% of DKA admissions involve patients with near-normal blood glucose levels, emphasizing the need for greater clinical awareness [2].

The use of sodium-glucose co-transporter 2 (SGLT2) inhibitors, such as empagliflozin, has emerged as a significant contributor to the development of EDKA [3,4]. These agents promote glycosuria, suppress insulin secretion, and increase glucagon levels - metabolic shifts that enhance ketone production even in the absence of marked hyperglycemia [5]. As a result, clinicians may overlook the diagnosis of DKA in patients on SGLT2 inhibitors who present without significant hyperglycemia.

Sepsis is defined as life-threatening organ dysfunction caused by a dysregulated host response to infection [6]. Sepsis is a common precipitant of DKA and EDKA, as systemic inflammation exacerbates insulin resistance, lipolysis, and ketogenesis [7,8]. Its biochemical overlap with EDKA such as acidosis, hypotension, and altered mental status can obscure the underlying cause of metabolic decompensation. When compounded by concurrent infections, the clinical presentation of EDKA can be obscured, delaying diagnosis and appropriate management.

Given that SGLT2 inhibitors like empagliflozin are increasingly prescribed due to their cardiovascular and renal benefits, clinicians must maintain a high index of suspicion for EDKA in patients presenting with unexplained metabolic acidosis, regardless of glucose levels [4,9]. This report presents a case of EDKA complicated by methicillin-resistant Staphylococcus aureus (MRSA) bacteremia, in which the diagnosis was confounded by overlapping features of sepsis and medication-induced metabolic changes. Unlike typical EDKA cases, this patient's presentation included persistent bradycardia and recent antibiotic use, features not commonly emphasized in previous reports. By exploring clinical reasoning, laboratory evaluation, and management strategy, this case underscores the diagnostic complexity of EDKA and the importance of maintaining a high index of suspicion in patients on SGLT2 inhibitors.

## Case presentation

**Figure 1:**
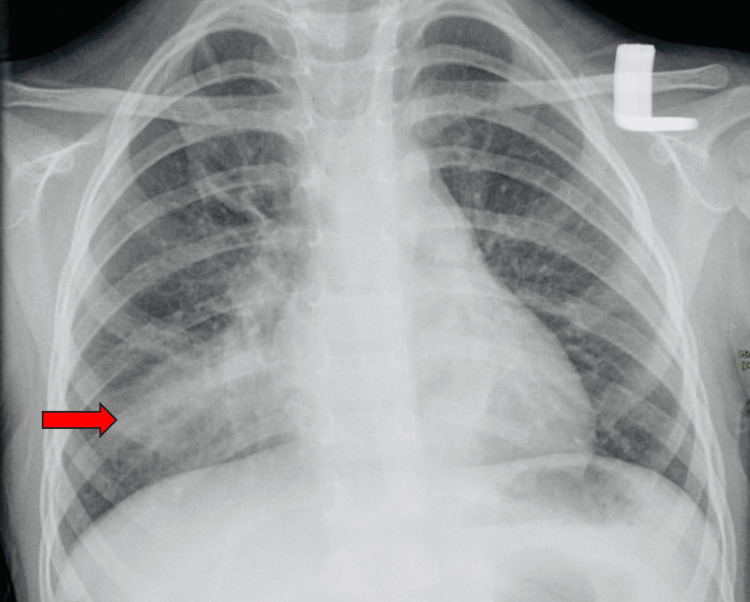
Patient chest imaging confirms the right lower lobe pneumonia.

A 69-year-old man with type 2 diabetes mellitus treated with metformin and empagliflozin (10 mg daily) presented to the emergency department with fever (38.5 °C), productive cough, and dyspnea. Physical examination revealed crackles in the right lower lung, tachycardia (110 bpm), and tachypnea (28 breaths/min). Chest imaging confirmed right lower lobe pneumonia (Figure 1), and empiric antibiotic therapy was initiated.

Within 24 hours, the patient developed hypotension (blood pressure 85/50 mmHg), oliguria, and altered mental status, meeting criteria for sepsis. He was transferred to the Intensive Care Unit (ICU) for further management. Initial laboratory evaluation (Table 1) revealed severe leukocytosis (WBC 36,000/μL), normal hemoglobin (15.6 g/dL), and platelet count of 375 × 10³/μL. Renal function showed blood urea nitrogen (BUN) 32 mg/dL and creatinine 1.1 mg/dL, indicating mild renal impairment. Liver enzymes were within normal limits, except for mildly elevated alkaline phosphatase (115 U/L). Serum bicarbonate was 8 mmol/L, with an anion gap of 31 and arterial pH of 7.14, confirming severe high anion gap metabolic acidosis. Arterial blood gas showed pCO₂ 23 mmHg and pO₂ 129 mmHg, reflecting partial respiratory compensation and supplemental oxygen use. Serum glucose was 209 mg/dL, indicating only mild hyperglycemia. Ketone levels were significantly elevated at 4.5 mmol/L, and hemoglobin A1C (HbA1C) was 8.0%, reflecting poor long-term glycemic control. In addition, lactic acid was within normal range (1.7 mmol/L), and procalcitonin was elevated (0.53 ng/mL), raising suspicion for bacterial infection. Electrolytes were unremarkable, with sodium at 137 mmol/L and potassium at 4.0 mmol/L.

**Table 1:** Laboratory Data on Admission (H) = High, (L) = Low, BUN = blood urea nitrogen, ALT = alanine aminotransferase, AST = aspartate aminotransferase, ALP = alkaline phosphatase

Laboratory Test	Result	Reference Range	Interpretation
White Blood Cells (WBC)	36,000/µL (H)	4,000–11,000/µL	Severe leukocytosis (infection)
Hemoglobin (Hb)	15.6 g/dL	13.5–17.5 g/dL	Normal
Platelets (Plt)	375 ×10³/µL	150–450 ×10³/µL	Normal
BUN	32 mg/dL (H)	7–20 mg/dL	Mild renal impairment
Creatinine (Cr)	1.1 mg/dL	0.7–1.3 mg/dL	Normal
Bilirubin	0.4 mg/dL	0.1–1.2 mg/dL	Normal
ALP	115 U/L (H)	40–129 U/L	Slightly elevated
ALT	13 U/L	7–56 U/L	Normal
AST	12 U/L	10–40 U/L	Normal
CO₂	8 mmol/L (L)	22–29 mmol/L	Severe acidosis
Anion Gap	31 (H)	8–16	Elevated (gap acidosis)
pH	7.14 (L)	7.35–7.45	Severe acidemia
pCO₂	23 mmHg	35–45 mmHg	Respiratory compensation
pO₂	129 mmHg (H)	80–100 mmHg	Oxygen therapy or hyperventilation
Hemoglobin A1c (HbA1c)	8% (H)	<7%	Poor glycemic control
Lactic Acid	1.7 mmol/L	0.5–2.2 mmol/L	Normal
Procalcitonin	0.53 ng/mL (H)	<0.1 ng/mL	Suggestive of bacterial infection
Glucose	209 mg/dL (H)	70–140 mg/dL	Mild hyperglycemia
Sodium	137 mmol/L	135–145 mmol/L	Normal
Potassium	4.0 mmol/L	3.5–5.0 mmol/L	Normal

Despite only moderate hyperglycemia, the findings confirmed high anion gap metabolic acidosis with ketosis, consistent with EDKA. The empagliflozin likely contributed to significant glycosuria, blunting the expected hyperglycemia typically seen in DKA.

**Figure 2:**
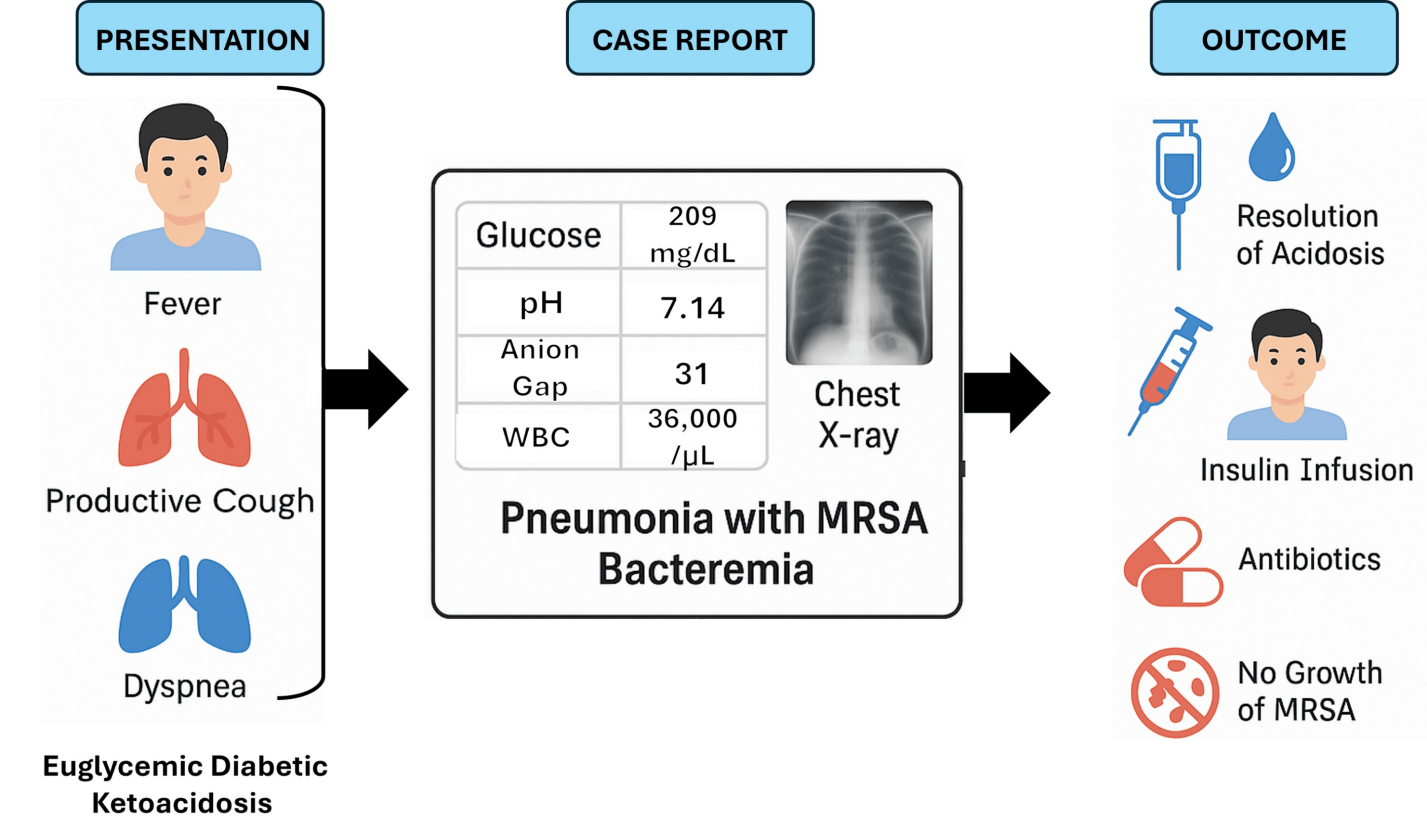
Clinical flowchart summarizing the patient’s presentation, diagnostic findings, interventions, and outcome. MRSA: methicillin-resistant Staphylococcus aureus

The patient was admitted to the ICU and managed according to established protocols for DKA. Management included intravenous fluid resuscitation with normal saline, continuous intravenous insulin infusion at 0.1 U/kg/h, and potassium supplementation, along with close monitoring of ketone levels. Empagliflozin, a likely precipitating factor, was discontinued immediately. Empiric antibiotic therapy with vancomycin (1g every 12 hours for four weeks) and ceftriaxone (1g every 24 hours for seven days) were initiated following consultation with the Infectious Disease team due to high-grade MRSA bacteremia of unknown source. Cardiology was consulted, and a transthoracic echocardiogram ruled out infective endocarditis or other cardiac pathology. The patient also developed clinically significant bradycardia, prompting a reduction in metoprolol dosage. Over the course of 48 hours, acid-base status normalized, and the patient gradually recovered from sepsis, ultimately leading to discharge after a 10-day hospitalization. Figure 2 summarizes the patient’s presentation, diagnostic findings, intervention, and outcome.

## Discussion

EDKA has become an increasingly recognized complication with the advent of SGLT2 inhibitors. The use of these agents has changed the epidemiology of DKA [1]. DKA is characterized by the triad of hyperglycemia, ketosis, and anion gap metabolic acidosis, with hyperglycemia (plasma glucose > 14 mmol/L) being a key diagnostic feature [10,11]. In contrast, EDKA is defined as ketoacidosis (pH < 7.3 or serum bicarbonate < 18 mmol/L) with near-normal plasma glucose or only mild hyperglycemia (11-14 mmol/L) with normal or mildly elevated plasma glucose (<250 mg/dL) [4,12]. Despite growing awareness, EDKA often remains underrecognized, particularly in critically ill patients, where acidosis may be attributed to other causes such as sepsis, renal dysfunction, or lactic acidosis [4,9,12]. In our case, the initial presentation with bradycardia, hypotension, leukocytosis, and fever prompted a working diagnosis of sepsis. However, the persistence of high anion gap acidosis despite appropriate fluid resuscitation and antibiotics should have raised earlier suspicion for EDKA, especially in the context of SGLT2 inhibitor use.

Several similar case reports describe delayed recognition of EDKA in patients treated with empagliflozin, where glucose levels remained deceptively normal despite significant metabolic acidosis [1-3]. This highlights the need to consider EDKA in any diabetic patient presenting with high anion gap acidosis, even if glucose levels are not markedly elevated. Early diagnosis of EDKA requires a high degree of clinical suspicion, especially in patients using SGLT2 inhibitors who present with metabolic acidosis of unclear origin. The key biochemical findings, including high anion gap metabolic acidosis, low to normal blood glucose levels, and positive serum ketones, should prompt consideration of EDKA, even when other severe conditions like sepsis are present [4,10]. According to the American Diabetes Association (ADA) and European Association for the Study of Diabetes (EASD), SGLT2 inhibitors should be discontinued during acute illness, including infections or any condition associated with decreased oral intake or volume depletion [13,14]. Management of EDKA involves discontinuing the precipitating agent, restoring intravascular volume, initiating insulin therapy, and correcting electrolyte imbalances. In our patient, timely ICU care, endocrine and infectious disease consultation, and surgical debridement of a previously unrecognized abscess contributed to favorable outcomes.

This case highlights the potential for missed or delayed diagnosis of EDKA in the presence of other severe conditions and emphasizes the importance of early recognition and a multidisciplinary approach. Clinicians should remain vigilant in considering EDKA in patients on SGLT2 inhibitors who present with unexplained acidosis, regardless of glucose levels. Educating healthcare teams about the atypical presentation of EDKA is crucial for prompt identification and intervention. Such vigilance can prevent delays in diagnosis and treatment, ultimately improving patient outcomes.

## Conclusions

This case underscores the importance of recognizing atypical presentations of EDKA, particularly in patients using SGLT2 inhibitors who present with concurrent infections such as sepsis. Beyond maintaining clinical suspicion, this case prompted our institution to implement a protocol advising temporary suspension of SGLT2 inhibitors during episodes of acute illness, including suspected infections. Additionally, it highlights the necessity for clinicians to maintain a high index of suspicion for EDKA in patients on SGLT2 inhibitors presenting with unexplained high anion gap metabolic acidosis, even when blood glucose levels are not significantly elevated. Early recognition, prompt discontinuation of the SGLT2 inhibitors, aggressive supportive care, and intensive insulin therapy are critical for successful management. Furthermore, patient and healthcare provider education regarding temporary withholding of SGLT2 inhibitors during acute illness is vital to prevent EDKA.
